# Operational method of reliability and content-validity analysis: Taking “trait-symptoms” screening of individuals at high-risk for OCD as an example

**DOI:** 10.1371/journal.pone.0232368

**Published:** 2020-05-12

**Authors:** Hongxiang Bao, Danmin Miao

**Affiliations:** Department of Medical Psychology, Fourth Military Medical University (Air Force Medical University), Xi'an, Shaanxi, China; Chinese Academy of Medical Sciences and Peking Union Medical College, CHINA

## Abstract

A well-designed self-reported scale is highly applicable to current clinical and research practices. However, the problems with the scale method, such as quantitative analysis of content validity and test-retest reliability analysis of state-like variables are yet to be resolved. The main purpose of this paper is to propose an operational method for solving these problems. Additionally, it aims to enhance understanding of the research paradigm for the scale method (excluding criterion-related validity). This paper used a study that involved screening of high-risk groups for OCD (Obsessive-Compulsive Disorder), conducted 5 rounds of tests, and developed scales, reliability, and validity analysis (using sample sizes of 496, 610, 600, 600 and 990). The operational method we propose is practical, feasible, and can be used to develop and validate a scale.

## Public health significance

Preventive screening of OCD among the general population has great practical significance due to its high prevalence, high costs, and diagnostic difficulty. Additionally, a well-designed self-reported screening scale is generally used in preventive, clinical, and personality studies. However, when using the scale method, there are few practical and quantitative methods for content validity analysis. Additionally, the test-retest reliability analysis of state-like variables, often conducted by traditional methods (such as the alpha coefficient (α)) is inappropriate and often incorrect. To overcome these problems, using screening of high-risk groups of OCD, the author explored and improved reliability and validity analysis. This study proposes an operational method for reliability and validity analysis that is practicable and feasible.

## Introduction

Initially, Spielberger proposed the concept of state-trait anxiety and state-trait depression; later, other scholars introduced state-trait anger [1]. Anxiety, depression, and anger are typically emotional and transitory in nature. However, obsessive-compulsive symptoms are not a purely typical emotions; therefore, the “state-OCD” may not be a widely acceptable concept. In 1980, Plutchik·R theorized that personality traits, defenses, and clinical symptoms are all derived from emotions and are all part of emotions [2]. Building on this, we analogously proposed the concepts of "symptom-OCD" and "trait-OCD". We also used a similar model to design a "symptom-OCD combined with trait-OCD " model to screen people at a high-risk for OCD because it is one of the most prevalent psychological and behavioral disorders (a military epidemiological study on mental diseases found that the 12-month prevalence rate of OCD (2.4%) is among the highest for single diseases). Due to its high prevalence, high costs, and diagnostic difficulty, OCD has become a significant public health concern [3]. Measuring OCD is complicated by its heterogeneity and high diagnostic comorbidity with other mental disorders [4]. However, a bigger reason is that most screening research focuses on OCD patients undergoing formal treatment rather than on the general population at high risk of developing OCD [5,6,7]. Given the circumstances, we attempted to define the population at high risk of OCD by taking into account its traits and symptoms, to effectively identify the high-risk population of OCD through potential temperament (dispositional) risk factors and severity of current symptoms. The traits may be viewed as relatively stable and enduring interindividual differences tending to react or behave in a particular way. In the current study, it included beliefs (obsessional beliefs, superstition) and OCD-metacognition, consisting of 4 screening scales. The symptoms have 2 meanings: One is that the subject has all the symptoms as described in DSM-5 (The diagnostic and statistical manual of mental disorders) for OCD, but the severity does not meet the diagnostic criteria; the other is that the subject has only some of the symptoms of OCD as described in the DSM. In our example of OCD, the symptoms included 2 scales.

It is a widely accepted fact that the self-reported scale is widely applied in clinical and scientific research as it is easy to use and has a straightforward rationale. More importantly, self-reporting is very effective for measuring psychological variables, especially personality and beliefs. However, the scale method needs to be improved in some areas; for instance, for content validity analysis, there are few effective, quantitative methods currently available. Although the content validity index (CVI) is an evaluation method to measure content validity, it assumes that the scale item already exists and that the scale structure is already determined [8]. However, before we develop any new scale there are insufficient items or even any pre-existing item. Therefore, the methods of CVI are not broadly applicable. Composite reliability is another important indicator in the evaluation of the quality of a scale, and possibly due to the lack of ready-made operational methodological tools, relevant studies are extremely rare. An additional issue that needs to be addressed here is that there are many state-like psychological constructs (e.g., state anxiety, state depression, state anger), and these are situational in nature and it may not be appropriate to evaluate their stability properties with the traditional test-retest reliability; however, the traditional reliability method is still widely used today. Although several studies related to test-retest reliability analysis are based on the LST (latent State-Trait model, the theory holds that human cognition, emotion and behavior are the result of the interaction of individual traits, situational characteristics and trait-situation interaction), few have provided specific and detailed procedures and thus lacked operability [9]. So far, the LST theory has not been used in OCD research. This paper proposes a practical operational method which is based on LST. This paper decomposes state latent variables into trait latent variables and situational latent variables (occasion factors) (**1.3.4 Methods on reliability analysis**).

In addition, this paper may facilitate understanding the scale method’s research paradigm, such as the issue of test-item selection, reliability, and validity analysis, especially the test-retest reliability analysis for state-like psychological attributes (criterion validity analysis is not discussed).

## 1.Methods

### 1.1 Measures and instruments

We performed a set of comprehensive screening tests on the general population, to identify individuals at greater risk of developing OCD in the future (OCD high-risk population). The 6 scales used in this study are.

The Obsessive Belief Questionnaire (OBQ) is a scale of OC-beliefs, including 44 items in 3 dimensions: a) responsibility/overestimation of threat (16 items), b) perfectionism/intolerance of uncertainty (16 items), and c) importance and control of thoughts (12 items) [10]. In this study, we translated the OBQ because there is strong evidence in literature that that this scale is valid for local Chinese college students, which may serve as an excellent measuring tool for assessing obsessive beliefs in the Chinese population [11].

The OCD-metacognition scale is a scale that we developed, on the basis of the early meta-cognitive theory of OCD [12], in combination with the cognitive self-consciousness scale [13]. This scale consists of 26 items in 3 dimensions: a) cognitive self-awareness (14 items), b) meta anxiety (7 items) and c) thought control (5 items).

The OCD superstition scale is a scale that we developed as Chinese and other foreign research studies have considered superstitious belief as an effective predictor for OCD [14]. This scale consists of 19 items in 3 dimensions: a) superstitious belief (8 items), b) superstitious motivation (7 items) and c) superstitious behaviors (4 items).

The NJRE (“not just right experience”) scale contains 19 items in 3 parts [15]. In recent decades, this scale was regarded as a powerful tool for screening patients with OCD [16]. However, few studies had been conducted on Chinese population. Considering the focus and need of this paper, we used the 10 items (these 10 items are the first part of the NJREs, specifically being used for presenting sample NJREs) of the NJRE scale.

We developed the OCD traits scale as no such screening scale currently exists. This scale is based on the DSM-5 temperamental risk factors related to OCD and was integrated with the two-way specification table results of this study. The scale has 54 items in 4 dimensions.

The OCD-symptoms (OC-symptoms) scale used in this paper was redeveloped from existing scales. There are a number of OCD-symptoms scales, such as the revised Padua Obsessive-Compulsive scale [17] (PI-R-41), Obsessive-Compulsive inventory OCI-CV [18], Revised Obsessive Intrusive Inventory (ROII) [19], FOCI-20 [20], the Yale Brown Obsessive-Compulsive Severity Scale Checklist (Y BOCS-SC- 10), etc. However, most of mentioned above scales either lack an adequate discriminant validity or are less specific to OCD. Therefore, the OCD-symptoms scale used in this paper was recompiled to obtain high specificity. Ultimately, the scale used in this paper ended up with 36 items in 2 dimensions.

### 1.2 Participants and samples

In this study, 5 rounds of tests were conducted, with the 3rd and 4th rounds being repeated measure designs. The first 2 rounds were designed to select the items, the 3rd and 4th were intended for test-retest reliability analysis, and the 5th was primarily for construct-related validity analysis. The participants and sample sizes of the 5 tests are as follows.

Round 1 was a pre-test and was conducted with a printed paper version. There were 496 participants, and most respondents (95%) were male. The participants ranged from 18 to 32 years of age (mean, 23.5 years; SD = 4.1). Years of military service ranged from 1 to 16 years, all being active military personnel and educational level was from junior high school to undergraduate.

Round 2 was also a printed paper test. A total of 610 participants (429 males, 181 females, 18–48 years of age) participated. Years of military service ranged from 1 to 30 years, all being active military personnel. Educational level was from junior high school to college graduate.

Rounds 3 and 4 (repeated measures design) were online tests. Two hundred college students underwent OBQ and OCD metacognition tests (120 males, 80 females, 20 to 26 years old, junior high school to undergraduate educational levels), 190 military undergraduates took the OCD superstition and NJRE tests (160 males, 30 females, 18 to 48 years old, 1 to 4 years of military service, junior high school to graduate level), and 180 first-year medical students tested for OCD traits and OCD symptoms (110 males, 70 females, 19 to 23 years old, junior high school to college freshman levels).

Round 5 was also conducted online, and 655 active military personnel were tested on 5 scales (530 males, 125 females, 18 to 46 years old, 2 to 21 years of military service, educational levels ranged from junior high school to undergraduate levels), and 998 active military personnel were tested on OBQ scales (880 males, 118 females, 18 to 24 years old, 1 to 2 years of military service, educational levels ranged from junior high school to undergraduate).

### 1.3 Methods and procedures

The statistical analysis used in this study included: correlation analysis, factor analysis (including exploratory factor analysis and confirmatory factor analysis),and discrete trend (for example, using standard deviation, items with large standard deviation will be retained.)(see **Table 1**); inter-rater reliability(see **Table 2**); ESEM (exploratory structural equation modeling) and CFA (see **Tables 3 and 4**); composite reliability, Cronbach alpha coefficient (α), test-retest reliability, etc. (see **Table 5**). Statistical software used in this study included SPSS and MPLU. The specific details above can be found in the corresponding section of this article.

**Table 1:** The final 54 items of OCD-trait screening scale after multiple selections (initially 80 items).

Item(type)	*n*	*SD*	Item-total correlation	Factor loading	*P*_H_-*P*_L_	Item-criterion correlation
**Dominance process**	48	Large (0.33–0.56)	Medium to large (0.38–0.68)	Medium to large (0.30–0.72)	All greater than 0.4	Good discrimination to the high and low score subjects
**Ideal point process**	6	Larger (0.45–0.56)	Small (0.06–0.30)	Small (0.06–0.27)	All Less than 0.3	Good discrimination to the high and medium score subjects

*P*_H_ and *P*_L_ refer to subjects who were ranked according to their scores, with the highest score of 27% and the lowest score of 27%.

**Table 2:** Two-way specification table used for the study of OCD-traits.

Survey content	Basic traits (Please use "1,2,3" to rate the importance in ())	Low-order traits	Importance rating (please tick with “√”)				Notes (if you think anything is missing from the list of OCD traits, please add below)
			Very important	important	Average	Not important	
OCD-traits	() Neuroticism	1.Lack of security					
		2.Excessive inhibition					
		3.Indecision, lack of confidence					
		4.Intolerance of uncertainty					
		5.Self-accusation					
		6.Introspective and rigid					
		7.Negative, pessimistic, cautious					
	() High sense of responsibility/morality	1.high sense of responsibility					
		2.high morality, strong sense of shame					
	() Implicit aggressive traits	1.Strong stress response and weak coping ability					
		2.Sensitive and fragile					
		3.iritability and quick temper					
		4.Emotional instability					
		5.Distrust and suspicion					
	() Pathological perfectionism	1.High standards or excessive strict					
		2.Self-evaluation relies too much on success					
		3.Higher self-criticism					
		4.Hate failures and flaws					
		5.Pathological obsession					
		6.Absolute symmetry, or absolute certainty					
		7. Mind control					

**Table 3:** Summary of fitting indexes of OCD-trait screening scale (models).

Model	χ^2^	*p* value	TLI	CFI	RMSEA (90% CI)	WRMR
Five-factor	1034.641	0.00	0.993	0.994	0.018(0.014,0.022)	0.719
Four-factor	1130.763	0.00	0.991	0.992	0.020(0.016,0.024)	0.783
Three-factor	1223.625	0.00	0.990	0.991	0.022(0.018,0.025)	0.846
Two-factor	1338.795	0.00	0.987	0.989	0.024(0.020,0.027)	0.923
Bifactor	1664.253	0.00	0.976	0.978	0.033(0.030,0.035)	1.128

**Table 4:** Results of the six scales’ analysis of construct-related validity.

scale	*n*	construct-related validity	Number of items
OBQ	998	Six-factor	36
OCD metacognition	655	Three-factor	26
OCD superstition	646	Three-factor	19
NJRE	647	One-factor	10
OCD traits scale	638	Three-factor	47
OCD symptoms scale	650	four-factor	36

**Table 5:** Six-scale reliability index (based on two types of models).

scales	CTT			LSTT		
	α	composite reliability	Test-retest reliability	*Con*	*Ospe*	*RC*
**OBQ**	0.865	0.885	0.429(sub-OBQ, 5 items)	0.462	0.032	0.494
**OCD-metacognition**	0.897	0.912	0.563(sub-OCD-metacognition, 5 items)	0.573	0.114	0.687
**OCD-superstition**	0.849	0.880	0.518(sub- OCD superstition, 3 items)	0.585	0.132	0.717
**NJRE**	0.838	0.882	0.252(10 items)	0.451	0.326	0.777
**OCD-traits scale**	0.959	0.963	0.658(sub-OCD-traits scale, 4 items)	0.592	0.002	0.594
**OC-symptoms scale**	0.914	0.926	0.599(sub-OC-symptoms scale, 4 items)	0.454	0.157	0.611

#### 1.3.1 Statistical methods on item selection

Item selection, conducted from a pool of items is a critical part of developing a scale. This paper adopted a multiple selection of items, such as discrete tendency [21], correlation coefficient, factor analysis from CTT (Classical Test Theory), item discrimination etc. (**Table 1**). Using this method, items with medium to high factor loading and large variances were retained. Additionally, items with small factor loading but large variations also had a chance of being retained, and the latter is often a more sensitive item whose supplementation may make the scale have greater discrimination and enable more accurate measurements, especially for medium level participants (this paper used the study on the OCD-trait scale as an example). Moreover, on further analysis, we found that these 2 kinds of items (items with large factor loading and items with small factor loading but large variance) were likely to be quite different in terms of the item response process, with the former matching a dominance model and the latter following an ideal point process [22] (**see Table 1**). In addition, the selection for the items should take full consideration of the item contents’ semantic appropriateness; for example, “I often feel unsafe around myself” and “When I walk, I like to go back and forth” are clearly not appropriate for an army camp environment (most of the participants in this study were soldiers, and military camps are perceived to be safe places according to the Chinese).

#### 1.3.2 Methods for content-related validity analysis

With regard to the content-related validity, previous analyses done were mostly qualitative. However, we employed a quantitative analysis method by basing it on a two-way specification table (**Table 2**), also known as a two-way checklist. The content validity analysis primarily focuses on the suitability and representativeness of test items, which is closely and directly related to the appropriateness of scale dimension (the top-level design of the scale) and sub-dimension design. This paper also used the OCD-trait scale as an example for the analysis. The OCD-trait items were mainly derived from 12 scales, such as the Morita neuroticism scale and the NEO-FFI-Neuroticism dimension.

In practice, we need a more systematic study of OCD-traits. Using DSM-5 and related literature [23], we preliminarily determined the top-level structure of Chinese conceptual OCD-traits, which ensured that the constructed scale contained core dimensions of the OCD-traits. Next, in practical applications, we used 3 levels of evaluators to rate the list. The 3 levels consisted of psychology graduates, psychology experts especially good in OCD, and senior psychiatrists with clinical experience. There were at least 3 people involved at each level. Finally, we compiled the results of the raters and finally obtained an inter-rater reliability.

It is worth mentioning that for specific calculation of the rater reliability of the 6 scales, different degrees of dependence were calculated, based on the results of the 3 levels of evaluators. For the OCD-traits (scale), we depended heavily on the ratings by the psychology experts and the professors; for the OCD-symptoms scale we relied more on the opinions of the psychiatrists. In addition, for the OBQ and OCD-metacognition scales, we used the 3-parts’ rating as mentioned previously. In this paper, we introduced only content-validity analysis of the lower-order traits and provided its analysis results and did the same for the item-level analysis.

#### 1.3.3 Methods on construct-related analysis

The factor analysis from SPSS and MPLUS were well combined to study construct-related validity. The main idea leveraged here was model competition (when several models fit the criteria, the simpler model is preferred). This is a data-based analytic perspective. In addition, our construct-related validity should also be analyzed from a theoretical perspective. This because we needed to verify whether the constructed concepts made theoretical sense. When several models are theoretically sound, we prefer the model that is most recognized and easily accepted. We still used the OCD-traits as an example (**Table 3**), but the results of the construct-related validity analysis of all 5 scales are also shown in **Table 4**. The goodness-of-fit statistics we selected were likelihood-ratio, chi-squares test, and its associated *p* value, 3 alternative measures of fit: an absolute fit index and 2 comparative fit indices. The absolute fit index consisted of the rooted mean square error of approximation (RMSEA) and its associated *p* value and confidence interval (CI). The 2 comparative fit indices consisted of the comparative fit index (CFI) and the non-normed fit index (NNFI), also known as the Tucker-Lewis Index (TLI). The fit of a model was judged by a small chi-square value relative to the model’s degree of freedom, RMSEA<0.05, CFI, and TLI>0.95 [24]. In addition, a special fitting index WRMR (weighted root mean square residual) for category variables is included (WRMR's cutoff is generally set at <1) [25].

#### 1.3.4 Methods on reliability analysis

In this paper, reliability was analyzed from 2 perspectives: internal consistency and test-retest psychometric properties. The reliability evaluation included the Cronbach alpha coefficient (*α*) and composite reliability. In addition, because the latent state-trait theory/model (LST) allows us to “examine” the effects of traits, situations, and their interactions at the same time, it and its 3 indexes (*Con*, *Ospe* and *RC*) were used to evaluate the scale’s retest performance. More importantly, for constructs that were strongly situation-dependent, showing high occasion specificities, high test–retest correlations could not be expected (and might not be appropriate). In contrast, the measures for reliability based on LST can still be perfectly reliable [26]. The In test-retest reliability analysis (based on third round testing and fourth round testing), the reliability indicators based on CTT and LST were both provided by us [27], and a comparison was additionally made between the 2 *Con* stands for consistency, reflecting the ratio of observed variance determined by traits. *Ospe* denotes occasion-specificity, representing the ratio of observed variance determined by situations and their interactions. Finally, *RC* indicates the reliability coefficient, the sum of *Con* and *Ospe*.

For calculating composite reliability (ρ) and the other 3 indices, please refer to formula (1), (2), (3) and (4). The LST model mentioned above can be expressed by the equation *Y_it_* = *λ_it_ T_it_*+*δ_it_*O_*t*_+*e_it_* **[28]**.

$$\rho =(\sum_{i=1}^{P}\lambda_{i}){}^{2}/[(\sum_{i=1}^{P}\lambda_{i}){}^{2}+p-\sum_{i=1}^{P}{\lambda_{i}}^{2}],$$ (1)

$$\mathrm{Con}\left(Y_{it}\right)=\lambda_{it}^{2}\mathrm{Var}(T)/\mathrm{Var}\left(Y_{it}\right),$$ (2)

$$Ospe\left(Y_{it}\right)=\delta_{it}^{2}\mathrm{Var}\left(O_{t}\right)/\mathrm{Var}\left(Y_{it}\right),$$ (3)

$$\mathrm{Rel}\left(Y_{it}\right)=[\lambda_{it}^{2}\mathrm{Var}(T)+\delta_{it}^{2}\mathrm{Var}(O_{t})]/\mathrm{Var}\left(Y_{it}\right).$$ (4)

(*λ_i_* represents the factor loading and *p* represents the number of items, number of indicators, under the CTT framework). In the LST theory, we usually need 2 indices for the observable variables (measurement indicators) *Y_it_*: the second index, *t*, refers to the *t*th occasion of measurement, whereas the first index, *i*, refers to the *i*th measurement of the person considered within occasion t, that’s, *Y_it_* represents the measurement indicator *i* on the measurement occasion *t, λ_it_* and *δ_it_* respectively represents the factor loading of the indicator on trait and occasion under the LST framework.)

### 1.4 Methods of handling missing values

The statistical analysis software used in this paper included SPSS 17.0, MPLUS 7.4. and EXCEL, EXCEL was used to manage the missing values. The missing values in this paper included 2 types. First, for the printed paper version of our test, if more than 10% of the test items were not answered, the record was considered invalid and deleted (a record is meant as any row of the EXCEL spreadsheet). Second, for a record in the online version of the test, if the participant was found to not have answered seriously or truthfully (for example, the answers to all items is “YES” or “NO” and the answer time was even less than the time it took to click directly without reading the item), the record was deemed to be invalid.

This study was approved by the Ethics Committee of the First Affiliated Hospital of the Fourth Military Medical University. In addition, all individual participants included in the study received verbal informed consent, that is, before the test, we informed all participants of the purpose of our test, and then asked all participants if they would agree to take the test voluntarily to ensure that all participants got informed consent. Besides, our study included no minors.

## 2.Result

### 2.1 Results of item selection

See Table 1

### 2.2 Results of validity analysis

This paper primarily focuses on the content-related validity and construct validity analysis. Criterion validity analysis is essentially a correlation analysis and was not covered in this paper.

#### 2.2.1 Content-related validity

See Table 2

Based on the score results of the above “**two-way specification table**”, the inter-rater reliability of the OCD-trait scale was 0.90, indicating a fairly satisfactory content validity, which proves that the method of two-way specification table used is practical and feasible.

#### 2.2.2 Construct-related validity

See Table 3 and Table 4

As shown in Table 3, except that the TLI and CFI parameters of the Bifactor model are relatively small and the WRMA index is greater than 1, the other 4 models fit well. Among them, the two-factor model is the simplest, but the three-factor model is easier to explain theoretically. After comprehensive consideration, the OCD-trait was finally determined as a three-factor model.

In terms of the relationship between the six scales, OCD-metacognition and OCD-traits were found to be more similar and related. In addition, there are many similarities between OC-beliefs and OC-symptoms. There was a lower correlation between NJRE and the other 4 scales, the same was true for OCD superstition.

### 2.3 Results of reliability analysis

The detailed reliability analysis results are shown in Table 5.

*Note*: During the retest experimental section, due to a certain number of subjects’ loss of follow up (for example, leaving the military for a few months), the sample size was insufficient. Therefore, we selected only 1 dimension (subscale) of each scale (except for NJRE) for calculating retest indexes (columns 4–7 of Table 5 above). Due to the small number of test items, the retest reliability values were relatively small (column 4 and 7).

By comparing the values of the 5th column, *Con*, with the value of the sixth *Ospe* columns (**Table 5)**, our research indicated that beliefs, OCD-metacognition, and traits tended to be more stable concepts. In contrast, the symptoms scale and NJRE seemed to feature a highly situational dependence. As can be easily found from the above **Table 5**, for the latter 2, *Ospe* accounts for a larger proportion in *RC*. Moreover, our studies of the relationships between the 6 scales showed that OCD-metacognition and OCD-traits seem to be more closely related, while OC-belief and OC-symptoms seemed to be more closely related (calculation for *Con*, *Ospe* and *RC* are in Table 5 is shown in the **S1 Appendix**).

## 3.Discussion

At present, the scale method is still the most economical and effective way to measure personality (traits) such as anxiety-related personality traits and neurotic traits. The main purpose of this paper was to provide readers with a more operational method of the scale research, namely, how to develop a psychological measurement scale, how to analyze the content-validity, how to conduct test-retest reliability analysis (especially for the symptomatic or emotional variables that are strongly situation-dependent), and how to select items in a proper manner.

These tasks may be critical for scale construction. For example, in terms of item selection, although previous approaches often relied on factor analysis or correlation coefficients alone, some useful items with small factor loading but larger variation might be ignored. Therefore, in order to keep as many useful items as possible in the scales, we adopted a multiple selecting strategy.

For the validity analysis, **Table 2** shows how the “two-way specification table”. Moreover, it shows the hierarchy of OCD-traits scale in detail. The results of previous studies [29], similar to the results of this paper, have showed that moral scrupulosity may be a relatively independent trait of OCD. Additionally, perfectionism has long been considered a cognitive variable, and a stable variable of traits-like. For the construct-related analysis of the OCD-trait scale, the results of factor analysis (**Table 3**) suggested that the two-factor model was more reasonable statistically, whereas the three-factor model is theoretically more acceptable. **Table 4** presented the construct-related validity results of other 5 scales and their fitting performance. It shows that the OBQ scale had a multidimensional tendency while the other 5 scales were unidimensional.

While analyzing the reliability of the scale, the coefficients under the two model frameworks of CTT and LST were provided (**Table 5)**. Traditional test-retest reliability analysis may not be suitable to measure constructs exhibiting a high situational dependence, because these kinds of variables are essentially situational. LST models, however, are appropriate to analyze of such attributes, because they take the fact that many psychological variables are affected not only by a stable person component (i.e., trait) into account but also by a systematic though unstable situational component. Furthermore, LST models have another function that allowed us to further determine whether a variable (i.e., OC-beliefs) is more likely to be stable trait or transient state affected by situation, which can help us better understand whether our constructed scale is appropriate from “a new perspective”. In other words, if a trait-like scale is constructed, but subsequent analysis finds that the proportion of *Ospe* in *RC* is too large, the scale may have inherent problems. This study found that OCD-traits and OCD-metacognition were more stable, while symptoms and beliefs were less stable. As for NJRE, the results of the data analysis suggested that it might be more symptomatic than the previously reported endophenotype [30]. Moreover, it did not appear to be suitable for screening high-risk groups in the Chinese military, because this study showed that the high-scoring individuals detected by the other 5 scales have high consistency, with the exception of NJRE. In addition, this study did not support its high specificity to OCD as described in the literature [31].

In conclusion, our set of screening scale has satisfactory content-validity, construct-related validity, and reliability to meet the requirements of psychometrics. Even more importantly, the item bank of OCD-high-risk screening scale was broad and large enough to cover all levels of the underlying traits, which also laid a substantial foundation for the next step: CAT (Computerized Adaptive Testing) implementation of screening. Moreover, the relationship between these 6 attributes on OCD need to be discussed further, such as whether there is a more complex moderating or mediating relationship between them. For example, literature has pointed out the mediating effect of perfectionism on the OC-symptoms [32]. Other findings highlighted that NJREs were a mediator of the relationship between IU and checking behaviors [33]. There are some areas of this paper that we wish to improve in future work. Such as the loss of participants, causing an insufficient sample size in the retest experimental study. This directly led to the inability to analyze the retest reliability at a full scale.

## Supporting information

S1 Appendix (DOCX)

S1 File (RAR)

## References

[1] Deffenbacher Jerry L., E. R. O. G. (1996). State-Trait Anger Theory and the Utility of the Trait Anger Scale. *Journal of Counseling Psychology*, 43(2), 131–148

[2] Plutchik R. (1980). Emotion: Theory, Research and Experience Volume 1 Theories of Emotion. ACADEMIC PRESS, INC.111 Fifth Avenue, New York, New York,10003

[3] Abramowitz J. S., & Deacon B. J. (2006). Psychometric properties and construct validity of the Obsessive–Compulsive Inventory—Revised: Replication and extension with a clinical sample. *Journal of Anxiety Disorders*, 20(8), 1016–1035. 10.1016/j.janxdis.2006.03.001 16621437

[4] ABRAMOWITZ J. (2008). Cognitive-behavioral therapy for OCD. *Clinical Psychology Review*, 28(2), 356 10.1016/j.cpr.2007.04.008 18060672

[5] Sattler A. F., Whiteside S. P. H., Bentley J. P., & Young J. (2018). Development and validation of a brief screening procedure for pediatric obsessive-compulsive disorder derived from the Spence Children's Anxiety Scale. *Journal of Obsessive-Compulsive and Related Disorders*, 16, 29–35. 10.1016/j.jocrd.2017.12.004

[6] Barzilay R., Patrick A., Calkins M. E., Moore T. M., Wolf D. H., Benton T. D, et al (2019). Obsessive-Compulsive Symptomatology in Community Youth: Typical Development or a Red Flag for Psychopathology? *Journal of the American Academy of Child & Adolescent Psychiatry*, 58(2), 277–286. 10.1016/j.jaac.2018.06.038 30738554

[7] Geller D. A., Doyle R., Shaw D., Mullin B., Coffey B., Petty C., et al (2006). A quick and reliable screening measure for OCD in youth: reliability and validity of the obsessive compulsive scale of the Child Behavior Checklist. *Comprehensive Psychiatry*, 47(3), 234–240. 10.1016/j.comppsych.2005.08.005 16635654

[8] Polit D. F., & Beck C. T. (2006). The content validity index: Are you sure you know what's being reported? critique and recommendations. *Research in Nursing & Health*, 29(5), 489–497. 10.1002/nur.20147 16977646

[9] Geiser C., & Lockhart G. (2012). A comparison of four approaches to account for method effects in latent state–trait analyses. *Psychological Methods*, 17(2), 255–283. 10.1037/a0026977 22309958 PMC3368086

[10] Myers S. G., Fisher P. L., & Wells A. (2008). Belief domains of the Obsessive Beliefs Questionnaire-44 (OBQ-44) and their specific relationship with obsessive–compulsive symptoms. *Journal of Anxiety Disorders*, 22(3), 475–484. 10.1016/j.janxdis.2007.03.012 17481852

[11] LEI Hui W. Q. W. Y. (2014). Reliability and Validity of the Chinese Version of the Obsessive Belief Questionnaire-87. *Chinese Journal of Clinical Psychology*, 22(02), 264–266(Chinese)

[12] Solem S., Myers S. G., Fisher P. L., Vogel P. A., & Wells A. (2010). An empirical test of the metacognitive model of obsessive-compulsive symptoms: Replication and extension. *Journal of Anxiety Disorders*, 24(1), 79–86. 10.1016/j.janxdis.2009.08.009 19748209

[13] de Bruin G. O., Muris P., & Rassin E. (2007). Are there specific meta-cognitions associated with vulnerability to symptoms of worry and obsessional thoughts? *Personality and Individual Differences*, 42(4), 689–699. 10.1016/j.paid.2006.08.015

[14] Moulding R., & Kyrios M. (2006). Anxiety disorders and control related beliefs: the exemplar of Obsessive–Compulsive Disorder (OCD). *Clinical Psychology Review*, 26(5), 573–583. 10.1016/j.cpr.2006.01.009 16647173

[15] Sica C., Bottesi G., Orsucci A., Pieraccioli C., Sighinolfi C.,… Ghisi M. (2015). “Not Just Right Experiences” are specific to obsessive–compulsive disorder: Further evidence from Italian clinical samples. *Journal of Anxiety Disorders*, 31, 73–83. 10.1016/j.janxdis.2015.02.002 25743760

[16] Sica C., Caudek C., Rocco Chiri L., Ghisi M., & Marchetti I. (2012). “Not just right experiences” predict obsessive–compulsive symptoms in non-clinical Italian individuals: A one-year longitudinal study. *Journal of Obsessive-Compulsive and Related Disorders*, 1(3), 159–167. 10.1016/j.jocrd.2012.03.006

[17] EZIO S. (1988). Obsessions and compulsions: The Padua inventory. *Behav Res Ther*, 26(2) 10.1016/0005-7967(88)90116-7 3365207

[18] Al F. E. H. J. (2002). The Obsessive-Compulsive Inventory: development and validation of a short version. *Psychol Assess*, 14(4), 485–496 12501574

[19] Purdon C., & Clark D. A. (1993). Obsessive intrusive thoughts in nonclinical subjects. Part I. Content and relation with depressive, anxious and obsessional symptoms. *Behaviour Research and Therapy*, 31(8), 713–720. 10.1016/0005-7967(93)90001-b 8257402

[20] Overduin M. K., & Furnham A. (2012). Assessing obsessive-compulsive disorder (OCD): A review of self-report measures. *Journal of Obsessive-Compulsive and Related Disorders*, 1(4), 312–324. 10.1016/j.jocrd.2012.08.001

[21] Hao Y., & Fang J. (2004). The Study of Statistical Methods Used for Item Selection. *Chinese Journal of Health Statistics*, 21(4), 209–211

[22] Carter N. T., & Dalal D. K. (2010). An ideal point account of the JDI Work satisfaction scale. *Personality and Individual Differences*, 49(7), 743–748. 10.1016/j.paid.2010.06.019

[23] Jiang W., & Zhang H. (2008). Swedish university scales of personality used among obsessive-compulsive disorder. *hanghai Archivesof Psychiatry*, 20(04), 206–210

[24] Karin S., Nina K., Helfried M., & Volker H. (2004). Decomposing person and occasion-specific effects: an extension of latent state-trait (LSI) theory to hierarchical LST models. *Psychological methods*, 9(2). 10.1037/1082-989X.9.2.198 15137889

[25] DiStefano C., Liu J., Jiang N., & Shi D. (2017). Examination of the Weighted Root Mean Square Residual: Evidence for Trustworthiness? *Structural Equation Modeling*: *A Multidisciplinary Journal*, 25(3), 453–466. 10.1080/10705511.2017.1390394

[26] Schermelleh-Engel K., & Keith N. (2004). Decomposing person and occasion-specific effects: an extension of latent state-trait (LSI) theory to hierarchical LST models. *Psychological Methods*, 9(2), 198–219 10.1037/1082-989X.9.2.198 15137889

[27] Steyer R., & Schmitt M. (1999). Latent state-trait theory and research in personality and individual differences. *European Journal of Personality*(13), 389–408. 10.1002/(SICI)1099-0984(199909/10)13:53.0.CO;2-A

[28] Yang Q., Yang B., & Wen Z. (2014). Computing Composite Reliability of A Unidimensional Tests by Using SPSS Software. *Chinese Journal of Clinical Psychology*, 22(03), 496–498(Chinese)

[29] Bowen R., Balbuena L., Baetz M., & Marwaha S. (2015). Mood instability in people with obsessive compulsive disorder and obsessive-compulsive personality traits. *Journal of Obsessive-Compulsive and Related Disorders*, 6, 108–113. 10.1016/j.jocrd.2015.07.003

[30] Sica C., Bottesi G., Caudek C., Orsucci A., & Ghisi M. (2016). “Not Just Right Experiences” as a psychological endophenotype for obsessive-compulsive disorder: Evidence from an Italian family study. *Psychiatry Research*, 245, 27–35. 10.1016/j.psychres.2016.08.005 27526314

[31] Ghisi M., Chiri L. R., Marchetti I., Sanavio E., & Sica C. (2010). In search of specificity: “Not just right experiences” and obsessive–compulsive symptoms in non-clinical and clinical Italian individuals. *Journal of Anxiety Disorders*, 24(8), 879–886. 10.1016/j.janxdis.2010.06.011 20627224

[32] Moretz M. W., & McKay D. (2009). The role of perfectionism in obsessive–compulsive symptoms: “Not just right” experiences and checking compulsions. *Journal of Anxiety Disorders*, 23(5), 640–644. 10.1016/j.janxdis.2009.01.015 19261436

[33] Bottesi G., Ghisi M., Sica C., & Freeston M. H. (2017). Intolerance of uncertainty, not just right experiences, and compulsive checking: Test of a moderated mediation model on a non-clinical sample. *Comprehensive Psychiatry*, 73, 111–119. 10.1016/j.comppsych.2016.11.014 27939647

