# Supplementary material for: Operational method of reliability and content-validity analysis: Taking “trait-symptoms” screening of individuals at high-risk for OCD as an example
Source: PLoS One. 2020 May 12;15(5):e0232368. doi: 10.1371/journal.pone.0232368 (PMC7217456; doi:10.1371/journal.pone.0232368)
Supplement: S1 Appendix — (DOCX) [file pone.0232368.s001.docx]

**Appendix:**

In order to get the values of *Con, Ospe* and *RC(Rel)* in model fitting, we hypothesized that the same measurement indicator (observed variable) was equally affected by traits (T) in repeated measures, while different measurement indicators were equally affected by situations (occasions, O) in the same time test. The procedure for LST fitting for OCD- trait subscale (4 items) was used as an example, and the program statement of MPLUS and the partial results after fitting were provided:

1. MPLUS program for LST of OCD-traits (4 items):

DATA: FILE IS C:\Users\bhx\Desktop\OCD-trait.dat;

VARIABLE: NAMES ARE a1-a4 b1-b4;

USEVARIABLES ARE a1-a4 b1-b4;

categorical= a1-a4 b1-b4;

ANALYSIS:

ESTIMATOR=WLSMV;

MODEL:

T1 BY a1* b1(1);

T1 by a2* b2(2);

T1 by a3* b3(3);

T1 by a4* b4(4);

O1 by a1* a2-a4(5);

O2 by b1* b2-b4(6);

T1 with O1@0 O2@0;

O1 with O2@0;

T1@1;

O1@1;

O2@1;

[O1@0 O2@0];

OUTPUT: sampstat STANDARDIZED MOD;

1. Partial results of model fitting are as follows:

MODEL RESULTS

Two-Tailed

Estimate S.E. Est./S.E. P-Value

T1 BY

A1 0.647 0.119 5.436 0.000

B1 0.647 0.119 5.436 0.000

A2 0.828 0.071 11.678 0.000

B2 0.828 0.071 11.678 0.000

A3 0.766 0.090 8.522 0.000

B3 0.766 0.090 8.522 0.000

A4 0.624 0.128 4.894 0.000

B4 0.624 0.128 4.894 0.000

O1 BY

A1 0.209 0.211 0.995 0.320

A2 0.209 0.211 0.995 0.320

A3 0.209 0.211 0.995 0.320

A4 0.209 0.211 0.995 0.320

O2 BY

B1 0.251 0.236 1.063 0.288

B2 0.251 0.236 1.063 0.288

B3 0.251 0.236 1.063 0.288

B4 0.251 0.236 1.063 0.288
